# High Doses of Inactivated African Swine Fever Virus Are Safe, but Do Not Confer Protection against a Virulent Challenge

**DOI:** 10.3390/vaccines9030242

**Published:** 2021-03-10

**Authors:** Estefanía Cadenas-Fernández, Jose M. Sánchez-Vizcaíno, Erwin van den Born, Aleksandra Kosowska, Emma van Kilsdonk, Paloma Fernández-Pacheco, Carmina Gallardo, Marisa Arias, Jose A. Barasona

**Affiliations:** 1 VISAVET Health Surveillance Center, Complutense University of Madrid, 28040 Madrid, Spain; jmvizcaino@ucm.es (J.M.S.-V.); alkosows@ucm.es (A.K.); 2 Department of Animal Health, Faculty of Veterinary, Complutense University of Madrid, 28040 Madrid, Spain; 3 MSD Animal Health, P.O. Box 31, 5830 AA Boxmeer, The Netherlands; erwin.van.den.born@merck.com (E.v.d.B.); emma.vankilsdonk@merck.com (E.v.K.); 4 European Union Reference Laboratory for ASF, Centro de Investigación en Sanidad Animal (INIA-CISA), Valdeolmos, 28130 Madrid, Spain; pacheco@inia.es (P.F.-P.); gallardo@inia.es (C.G.); arias@inia.es (M.A.)

**Keywords:** African swine fever, inactivated virus, vaccine trial, domestic pigs

## Abstract

African swine fever (ASF) is currently the major concern of the global swine industry, as a consequence of which a reconsideration of the containment and prevention measures taken to date is urgently required. A great interest in developing an effective and safe vaccine against ASF virus (ASFV) infection has, therefore, recently appeared. The objective of the present study is to test an inactivated ASFV preparation under a vaccination strategy that has not previously been tested in order to improve its protective effect. The following have been considered: (i) virus inactivation by using a low binary ethyleneimine (BEI) concentration at a low temperature, (ii) the use of new and strong adjuvants; (iii) the use of very high doses (6 × 10^9^ haemadsorption in 50% of infected cultures (HAD_50_)), and (iv) simultaneous double inoculation by two different routes of administration: intradermal and intramuscular. Five groups of pigs were, therefore, inoculated with BEI- Pol16/DP/OUT21 in different adjuvant formulations, twice with a 4-week interval. Six weeks later, all groups were intramuscularly challenged with 10 HAD_50_ of the virulent Pol16/DP/OUT21 ASFV isolate. All the animals had clinical signs and pathological findings consistent with ASF. This lack of effectiveness supports the claim that an inactivated virus strategy may not be a viable vaccine option with which to fight ASF.

## 1. Introduction

African swine fever (ASF) is currently the major concern of the global swine industry and is one of the greatest problems for global animal health. ASF is a viral disease that affects domestic pigs and wild boar. At the beginning of outbreaks in ASF-free areas, the disease is highly lethal and is characterized by fever, signs of hemorrhage and sudden deaths 4–9 days after infection [1,2].

No vaccine or treatment is available to control the disease, signifying that control measures only depend on early detection in the field and rapid laboratory diagnosis, followed by the implementation of strict sanitary measures [3]. These strict sanitary measures include the removal of infected and in-contact animals and the establishment of national and international trade barriers, all of which have devastating economic consequences, mainly in countries with a large amount of swine production [4,5].

Since its re-introduction into Europe through Georgia in 2007, ASF virus (ASFV) has spread uncontrollably to numerous European countries, including Armenia (2007), Azerbaijan (2007), the Russian Federation (2007), the Ukraine (2012), Belarus (2013), Lithuania (2014), Poland (2014), Latvia (2014), Estonia (2014), Moldova (2016), Romania (2017), the Czech Republic (2017), Bulgaria (2018), Hungary (2018), Belgium (2018), Slovakia (2019), Serbia (2019), Greece (2020), and recently, Germany (OIE WAHIS, 2020) [6]. With the exception of the Czech Republic, which was declared ASF free in April 2019, and recently Belgium in November 2020, ASFV has continued to spread through the EU in 2020, mainly within wild boar populations. Cases of ASF in wild boar have continued to be reported in Bulgaria, Estonia, Germany, Hungary, Latvia, Lithuania, Poland, Romania, and Slovakia since August 2020. In some of these countries, such as Estonia, Germany or Hungary, ASF is reported only in wild boar populations but not in domestic pigs. The highest monthly totals of wild boar cases are continually reported in Hungary, Poland and Romania.

ASF was first detected in Asia in August 2018. This occurred in China, the largest pig producer in the world, with a population of approximately 400 million pigs [7]. The spread of ASFV throughout the Asian continent has been faster than in Europe and has affected 11 other Asian countries since 2019 (Mongolia, Vietnam, Cambodia, Hong Kong, North Korea, Laos, Myanmar, the Philippines, South Korea, Timor-Leste and Indonesia), India in 2020, and Malaysia in 2021 [6].

The tendency to become endemic in affected areas and the continuous spread to ASF-free areas demonstrates that the control measures taken to date are not sufficient, thus making it clear that it is necessary to reconsider and improve the ASF control and prevention programs. The greatest challenge of ASF control is the development of a safe and effective vaccine [8], hence the recent intensive research efforts related to the development of an ASF vaccine [9,10,11,12,13,14,15]. These initiatives have, for the last two years, been widely supported in a cost-effective manner by public authorities (e.g., the EU "VacDIVA” H2020 project, 862874) and private companies in order to accelerate the development of a vaccine against the ASFV strains currently circulating in Eurasia.

Numerous studies employing vaccine candidates have been carried by means of classic and innovative strategies, using different isolates, doses and administration routes [16]. However, despite the research efforts, no vaccine candidate that is completely safe and effective and that can be made available in the near future has yet been found.

The vaccination trials carried out to protect animals from ASF using natural live attenuated vaccines (OURT88/3, NH/P68, Lv17/WB/Riel) have, to date, attained the most promising results as regards effectiveness. This is because the immune response they provide against virulent ASFV is characterized by the absence of clinical signs and a reduction in or absence of viremia [13,17,18,19]). However, these types of vaccine candidates have been associated with safety concerns, not only because of their infectious nature and the possibility of reversion to virulence [20], but also because the appearance of chronic forms of the disease in some domestic pigs has historically been related to attenuated ASFV isolates [21,22].

This has, therefore, led to a great interest in researching other types of vaccines, such as genetically modified live attenuated virus. These vaccine candidates have, to date, proven to be effectively protective against parental and homologous viruses, but have a lack of protection against heterologous viruses [9,11,17,23]. Moreover, the virulence-linked genes are not yet well known, and the genetic modification of genes may affect the ability of the virus to induce a protective host response [17].

Inactivated or subunit vaccine prototypes are interesting from a safety point of view. Unfortunately, they have not yet been shown to be completely effective [24,25]. Several studies on subunit vaccine strategies have been conducted, including those related to antigen-, DNA-, and virus vector-based vaccines. These studies have demonstrated partial [12,14,26,27] or no protection against the challenge [10,28,29]. The key aspect as regards developing successful subunit vaccines is that of knowing the specific antigens linked to the induction of a host protective response, and these are still unknown [25].

Fewer studies have been conducted in the case of inactivated vaccine strategies. Most of those that have date back more than 35 years [30,31], and a protective effect has been seen in only some cases [32,33]. The most recent study with inactivated ASFV took place in 2014 and tested it with new modern adjuvants [24]. This approach did not appear to improve the efficacy, as no protection was observed in vaccinated animals.

However, other strategies that have still not been tested could improve the efficacy of inactivated ASFV preparations, such as the use of very high antigen doses. High doses of inactivated virus have been correlated with an increase in the immune response and protection, as has, for example, occurred with the inactivated bovine viral diarrhea virus (BVDV) vaccine [34], the inactivated poliovirus type 2 vaccine [35] and the influenza vaccine [36].

The objective of the present study is to test the safety, the ability to induce an antibody response and the ability to confer protection against a virulent ASFV isolate of an inactivated vaccine candidate, with a particular focus on a vaccination strategy that has not been previously tested with inactivated ASFV preparations. The following were considered: (i) virus inactivation by a low binary ethyleneimine (BEI) concentration at a low temperature [37], (ii) the use of new and strong adjuvants; (iii) the use of very high doses (6 × 10^9^ haemadsorption in 50% of infected cultures (HAD_50_)); and (iv) simultaneous double inoculation by two different routes of administration: intradermal and intramuscular.

## 2. Materials and Methods

### 2.1. Animals

The experiment was performed under biosafety level 3 conditions at the VISAVET Centre facilities at the Complutense University of Madrid. The experiment was carried out using 12 six-week-old domestic pigs weighing 10–15 kg, which were obtained from a commercial pig farm in Burgos, Spain. These piglets had not been vaccinated against any infectious disease and were antibody negative for the following pathogens: ASFV, classical swine fever virus, porcine reproductive and respiratory syndrome virus, *Mycoplasma pneumoniae*, and porcine circovirus type 2. Animal care and procedures were performed by following the guidelines of good experimental practices according to European, national, and regional regulations and under the supervision and approval of the Ethics Committee of Madrid (reference PROEX 004/19). The approved protocol included a detailed description of the efforts made to provide environmental enrichment and avoid the animals’ unnecessary suffering, including humane endpoints and the guidelines for euthanasia.

### 2.2. Virus

The virulent and haemadsorbing (HAD) genotype II Pol16/DP-OUT21 ASFV was isolated at the European Union Reference Laboratory for ASF, Centro de Investigación en Sanidad Animal, Instituto Nacional de Tecnología Agraria y Alimentaria (INIA-CISA) after 3 passages in porcine blood monocytes (PBM) from a domestic pig slaughtered during the outbreak that occurred in Leśna, Poland in September 2016. The Pol16/DP/OUT21 ASFV isolate was inactivated for the preparation of the vaccine and was subsequently used, without inactivation, for the challenge. Titrations of the ASFV stock were performed in PBM to monitor the end-point dilution, and viral titers were determined as the amount of virus causing haemadsorption in 50% of infected cultures (HAD_50_/mL).

### 2.3. Inactivation of Pol16/DP/OUT21 ASFV

For immunization purposes, the virus was grown on PBMs to high titers (1.47 × 10^12^ HAD_50_/mL) and the process included a freeze–thaw cycle to release the virus from the cells. After the freeze–thaw cycle, the virus suspension was purified. First, it was centrifuged at low-speed (10 minutes at 2000× *g* at 4 °C) to sediment the cellular debris; then, the supernatant containing the virus particles was filtered (MINISART filters of 0.45 microns) to remove cellular contaminants. Additionally, before the inactivation with BEI, these virus particles were centrifuged for 5 minutes at 10,000× *g* at 4 °C to remove any remaining cellular debris. A stock of 0.545 M BEI was prepared by mixing 1.09 M 2-bromo-ethylamine hydrobromide (BEA) with an equal volume of 1.91 N NaOH, followed by incubation at 37 °C for 30 minutes. This stock was used immediately in order to inactivate the virus suspension at a concentration of 5 mM BEI for 24 h at room temperature. The remaining BEI was neutralized with 10 mM sodium thiosulphate for 2 hours at room temperature before the inactivated virus was stored at −80 °C until further use.

Inactivation with BEI has minimal effect on protein and the ASFV maintains its antigenic capacity, as previously described [37,38]. In addition, antigen ELISA showed that ASFV p72 protein was still intact after BEI inactivation. The absence of an infectious virus was verified after three subsequent passages, during which the PBMs infected with the inactivated Pol16/DP/OUT21 ASFV were sub-cultured up to three times and further subjected to the OIE Universal Probe Library (UPL) real-time PCR (OIE 2019). The results indicated that a complete inactivation had been obtained.

### 2.4. Study Design

Five groups of pigs (*n* = 2) were simultaneously inoculated with the inactivated vaccine preparations by both intramuscular and intradermal routes, and each route inoculation was at a dose of 6 × 10^9^ HAD_50_. Four of them were formulated in different adjuvants: G1, MF59^®^ (an oil-in-water adjuvant); G2, Silica oil (an oil-in-water adjuvant with silica); G3, mGNE (a type of incomplete Freund’s water-in-oil adjuvant); G4, Montanide^TM^ ISA201 VG (a water-in-oil-in-water adjuvant from SEPPIC)), while the remaining preparation did not contain an adjuvant (G5; Table 1). The intradermal administration was performed with the IDAL needle-free injector (MSD-AH) [39]. The remaining two animals were maintained naïve as the control group.

Four weeks after prime inoculation, the animals were boosted with the cognate adjuvant using the same dose and administration routes. Two weeks later, all the groups were intramuscularly challenged with 10 HAD_50_ of the highly virulent Pol16/DP/OUT21 virus isolate.

EDTA-blood and serum samples were taken once a week during the vaccination period, i.e., the time between the day of the prime inoculation and the day before the challenge. EDTA-blood and serum samples were also taken at 4, 7 and 10 days after the challenge.

### 2.5. Clinical Sign Monitoring

The animals’ health status was expressed in terms of a quantitative clinical score (CS) specific to ASFV infection in domestic pigs, as previously described by Gallardo et al. (2017) [40]. Animal observations took place daily through the use of video camera monitoring (recording 24 hours a day) and veterinary visits. These means were employed to record and detect all clinical signs daily, with the exception of temperature. Rectal temperature was measured once a week during the immunization period and daily during the challenge period. Fever was defined as a rectal temperature greater than or equal to 40 °C.

The daily observation of the animals allowed us to take early action when required in order to avoid their unnecessary suffering. Euthanasia was performed by following the humane endpoints described by Gallardo et al. (2018) [41] if the accumulative CS was >18, or the animals showed any of the following severe clinical signs for more than two consecutive days: severe anorexia, recumbency, respiratory or digestive symptoms. Animals that were suffering unacceptably, according to veterinary criteria, without reaching the humane endpoint were also euthanized.

The protocol followed for euthanasia was previously described by Barasona et al. (2013) [42]: an injection of T61^®^ (Intervet, Madrid, Spain) via the intravenous route followed by anesthesia by the intramuscular injection of a combination of tiletamine–zolazepam (Zoletil 100 mg/mL, Virbac, France, target dose 3 mg/kg) and medetomidine (Medetor, Virbac, France, target dose 0.05 mg/kg).

### 2.6. Necropsy and Tissue Sample Collection

Necropsy was performed on all the animals in order to detect any pathological lesions compatible with ASFV infection. Tissue samples of spleen, kidney, liver, brain, bone marrow, a pool of first barrier organs/peripheral lymph nodes (submandibular, retropharyngeal, prescapular, and inguinal), abdominal cavity organs (urinary bladder, renal lymph node, gastrohepatic lymph node, and mesenteric lymph node), and thoracic cavity organs (heart, lung, and mediastinal lymph node) were collected from each necropsied animal.

### 2.7. Laboratory Investigations

The EDTA-blood and tissue samples were investigated for the presence of the ASFV genome by employing real-time PCR (qPCR), following the protocol described by King et al. (2003) [43]. The results were expressed in cycle of quantification values (C_q_; equivalent to cycle threshold, CT). Viral DNA was extracted from EDTA-blood using the High Pure PCR Template Preparation Kit (Roche®) as described by the manufacturer.

The serum samples were investigated for the presence of ASFV antibodies by employing commercial ELISA assays to detect specific antibodies against either p72 (INGEZIM PPA Compac, Ingenasa, Madrid, Spain) or p32 (ID Screen® African Swine Fever Competition, IDVET, Grabels, France), following the protocol described by the manufacturers.

### 2.8. Statistical Analysis

The statistical analyses were conducted using R 3.5.0 [44] and SPSS 20 (IBM, Somar, NY, USA). The Kaplan–Meier survival curve and the Mantel–Cox log-rank test were used, respectively, to visualize the probability of death and to test for significant survival differences among all groups of vaccinated and control. The Kruskal–Wallis test was used to compare the differences between the clinical score, the temperature and the qPCR C_q_ from blood and tissues of the vaccinated and control groups. Pairwise comparisons were made using the Wilcoxon rank-sum test, with the *pairwise.wilcox.test* function from the R-package pgirmess [45]. Correlations between rectal temperatures and qPCR results from blood samples were analyzed using the Spearman rank test. A *p*-value < 0.05 was considered statistically significant.

A general linear model was performed to assess the effects of the study group (the five different vaccinated groups or the control group) and the days that had accumulated during the development of clinical signs for the last individual clinical score recorded. Visual inspections of the residual plots were used to confirm the assumptions of the model [46].

## 3. Results

The only side effect observed after vaccination was a local erythema at the point of intradermal inoculation. This lesion was observed more markedly in the Group 3 (mGNE) and Group 4 (Montanide ISA 206 VG) animals. The skin reaction was observed after both the prime and boost vaccinations. No other side effect was observed during the 42-day vaccination period.

None of the animals showed signs of viremia during the vaccination period, and none of them showed signs of a positive antibody response against ASFV p32. Only one animal in Group 1 had a doubtful and then a positive antibody response against ASFV p72 at day 35 after prime vaccination (7 days after boosting) and day 42 after prime vaccination (14 days after boosting), respectively.

**Figure 1:**
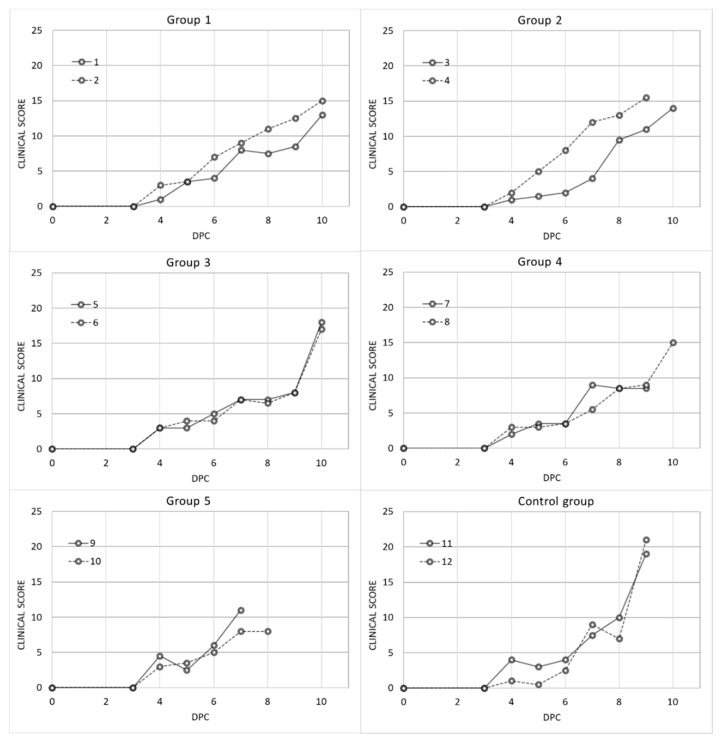
Individual post-challenge clinical scores of domestic pigs from the different vaccinated groups: Group 1 (Pol16-inactivated, MF59), Group 2 (Pol16-inactivated, Silica oil), Group 3 (Pol16-inactivated, mGNE), Group 4 (Pol16-inactivated, Montanide ISA201), group 5 (Pol16-inactivated), and the control group.

After the challenge, both the vaccinated and control groups had ASF-compatible clinical signs. All the clinical signs were recorded daily as a quantitative CS. There were no statistically significant differences among the CS obtained for the different study groups (Kruskal–Wallis test, *p* = 0.8125; see Figure 1).

**Figure 2:**
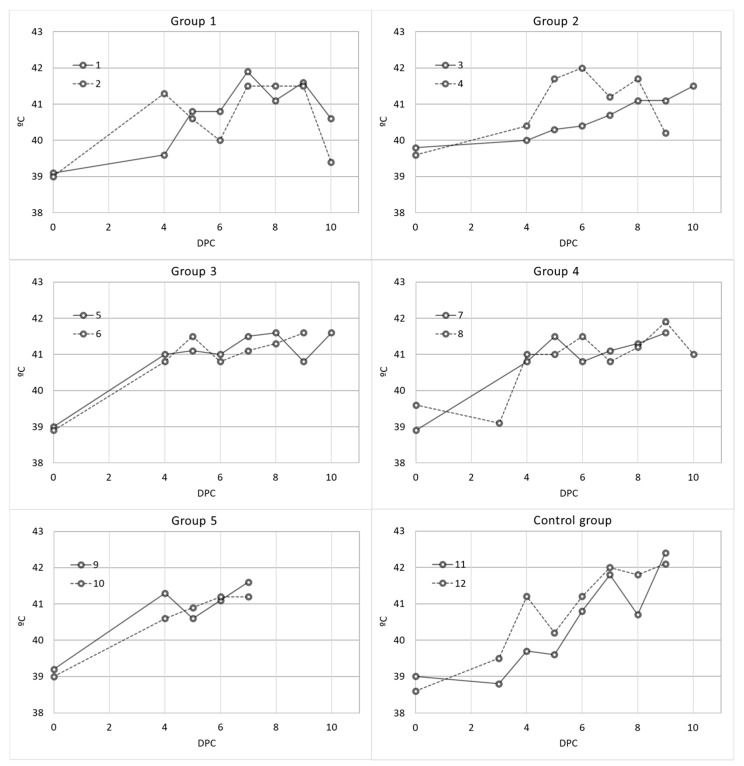
Individual post-challenge temperatures of domestic pigs from the different vaccinated groups: Group 1 (Pol16-inactivated, MF59), Group 2 (Pol16-inactivated, Silica oil), Group 3 (Pol16-inactivated, mGNE), Group 4 (Pol16-inactivated, Montanide ISA201), Group 5 (Pol16-inactivated), and control group.

From day 5 ± 1 post-challenge, nonspecific clinical signs such as fever, lethargy and anorexia were observed in both the vaccinated and control animals. The first symptom observed in all the animals from the different study groups was fever, starting from day 4 ± 1 post-challenge. The animals in all the groups had temperatures higher than 40.5 °C from day 7 post-challenge (Figure 2). There were no statistically significant differences among the rectal temperatures obtained for the different study groups (Kruskal–Wallis test, *p* = 0.994).

From day 7 ± 1 post-challenge, the animals developed more specific clinical signs, such as skin marks with erythema and a slight swelling with weakness in the hindquarters. These symptoms were observed in all the study groups with the exception of Group 5, whose members did not develop swelling and walking difficulties. From day 9 ± 1 post-challenge, other clinical signs, such as respiratory distress and diarrhea, were successively observed in the animals, leading to euthanasia for animal welfare reasons, with the exception of the two vaccinated animals from Group 5 and one animal from Group 4.

The last value of the clinical score, which was assessed by employing the linear model, was higher in the control group when compared to all the vaccinated groups (β = 6.67; d.f. = 5; *p* < 0.05), taking into account the factor of the days that had accumulated during the development of clinical signs, since the longer this time period is, the greater the clinical score expected.

**Figure 3:**
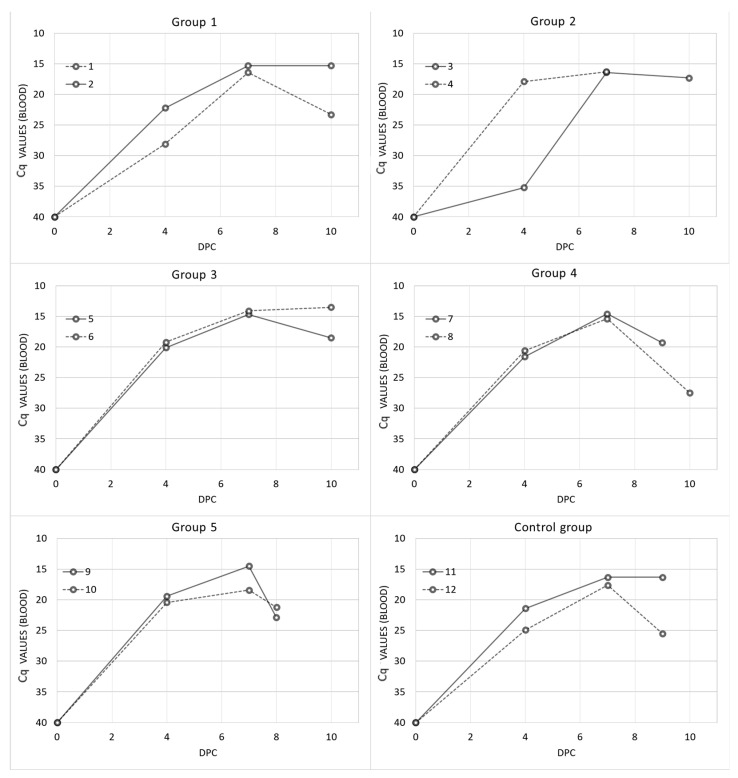
Individual post-challenge viremia results expressed in qPCR cycles of quantification values (C_q_) of domestic pigs from the different vaccinated groups: Group 1 (Pol16-inactivated, MF59), Group 2 (Pol16-inactivated, Silica oil), Group 3 (Pol16-inactivated, mGNE), Group 4 (Pol16-inactivated, Montanide ISA201), Group 5 (Pol16-inactivated), and the control group.

The onset of an increase in rectal temperature coincided with the detection of the ASFV genome in blood (Spearman rank test: r = 0.59, *p* < 0.01). All the animals in the different study groups were positive to the ASFV genome on day 4 post-challenge, starting with C_q_ = 25 ± 4 (Group 1), C_q_ = 27 ± 12 (Group 2), C_q_ = 20 ± 1 (Group 3), C_q_ = 21 ± 1 (Group 4), C_q_ = 20 ± 1 (Group 5) and C_q_ = 23 ± 3 (Control group; See Figure 3). The blood samples remained positive until the last sampling. No significant differences were observed among the C_q_ values obtained from the different study groups (Kruskal–Wallis test, *p* = 0.9146).

By day 10 post-challenge, all the animals had succumbed to the disease. The two animals from Group 5 and one animal from Group 4 were found dead on days 7, 8 and 9 post-challenge, respectively. The remaining animals were euthanized upon reaching the pre-established humane endpoint on days 9 and 10 post-challenge. The animals found dead had attained a lower level of the clinical score before succumbing to the disease (Mann–Whitney U test, *p* < 0.01).

**Figure 4:**
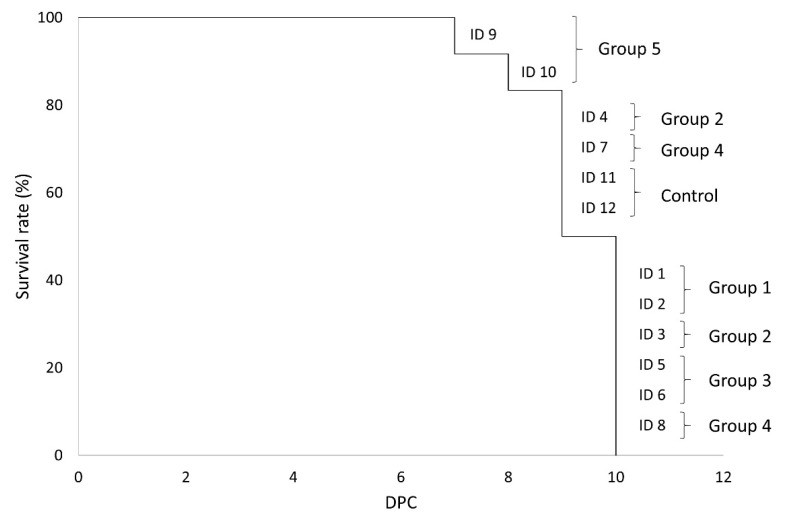
Kaplan–Meier survival curve of vaccinated and control domestic pigs subsequently challenged intramuscularly with the virulent Pol16/DP/OUT21 ASFV. DPC: days post-challenge.

There were statistically significant differences among the groups as regards survival time (Mantel–Cox, χ2 = 16.88, 5 d.f.; *p* = 0.005). The animals from Group 5 succumbed one and two days before those in the control group, while the animals from Groups 1 and 3 reached the humane endpoint after the control group (Figure 4).

**Figure 5:**
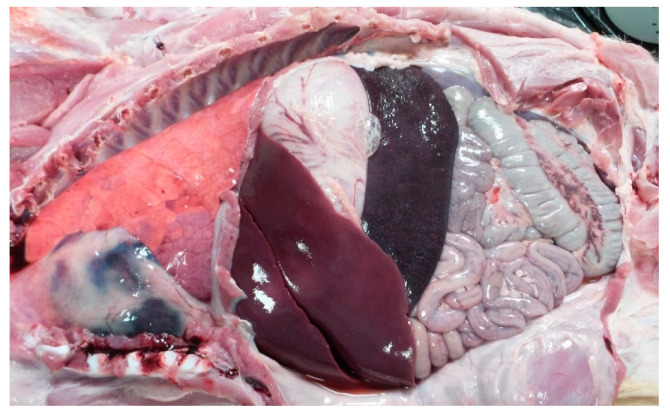
View of thoracic and abdominal cavities of a vaccinated pig (group 2, ID 3) subsequently challenged intramuscularly with the virulent Pol16/DP/OUT21 ASFV. Hepatomegaly, splenomegaly and ascites are evident.

During post-mortem evaluation, all the vaccinated and control animals had pathological lesions consistent with ASF. All the animals developed splenomegaly, lymphadenomegaly, hepatomegaly and an accumulation of yellowish to reddish fluid in the abdominal cavity (ascites), thorax (hydrothorax), and pericardial sac (hydropericardium; see Figure 5). Other intermittent observed pathological lesions were hemorrhagic lymphadenitis, congestion in the kidneys, perirenal edema, and hemorrhages in the stomach and intestines.

**Figure 6:**
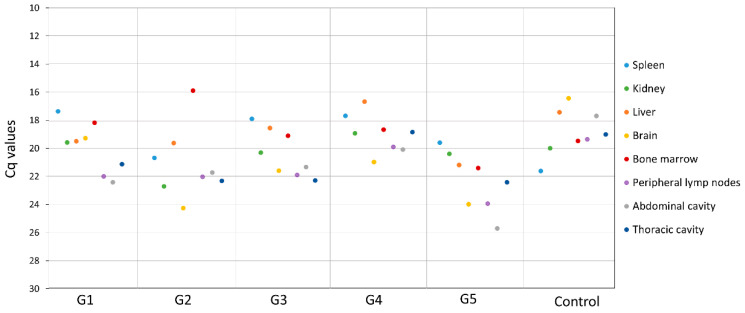
Mean of the cycles of quantification values (Cq) obtained by qPCR from the spleen, kidney, liver, brain, bone marrow, peripheral lymph nodes (submandibular, retropharyngeal, prescapular, and inguinal), abdominal cavity organs (urinary bladder, renal lymph node, gastrohepatic lymph node, and mesenteric lymph node) and thoracic cavity organs (heart, lung, and mediastinal lymph node) of the different study groups.

In line with the clinical course and post-mortem evaluation, all tissues from all the vaccinated and control animals tested positive in qPCR (see Figure 6). There were no significant differences among the different groups of study for any of the tissues tested or group of related tissues (Kruskal–Wallis, *p* > 0.226).

## 4. Discussion

The objective of this study was to assess the application of a mild BEI-inactivated ASFV preparation with a high antigen dose, the simultaneous use of intradermal and intramuscular routes of administration and a vaccination schedule with a long-time interval between prime and boost (i.e., 4 weeks), along with the use of strong modern adjuvants to enhance the efficacy of inactivated ASF vaccines. Despite these efforts, the results described above indicate that the BEI-inactivated ASFV preparations used under the conditions of this experiment were not able to induce effective protection against a lethal challenge. The results obtained are in line with those previously observed a considerable amount of time ago with other inactivated ASFV preparations [24,30,31,32,33]. Mild BEI inactivation conditions were chosen such that protective epitopes would remain preserved [37], however it is important to note that it was not confirmed that all the antigens were still intact after inactivation.

During the vaccination period, prior to challenge, the only side effect observed was the local erythema at the point of intradermal inoculation. Therefore, it is consequently possible to conclude that the simultaneous intramuscular and intradermal administration of a total of four inoculations of 6 × 10^9^ HAD_50_ of BEI-inactivated ASFV is safe for domestic pigs. This is in line with the fact that none of the animals showed viremia during this period, as might be expected. The local erythema was likely owing to the strong adjuvants used, mainly mGNE and Montanide ISA 206 VG because the lesion was more markedly in those groups of study (3 and 4). In addition, it is possible that some soluble porcine proteins have remained within the inactivated preparation, since the purification procedure performed (centrifugation and filtration) only removes membranous cellular debris and large protein inclusions. These soluble porcine proteins could have caused some effect on the local reaction in the intradermal inoculation point and could even have caused immunomodulatory effects that influenced the immune responses against ASFV.

Only one animal from a vaccinated group (Group 1) had a positive antibody response against ASFV, specifically against the p72 antigen, during the vaccination period. Vaccine efficacy failure was not necessarily triggered by this lack of antibody response, since several studies have demonstrated that the antibodies generated in ASFV infection are not neutralizing. Furthermore, previous studies have observed that animals that did not have ASFV-specific antibodies during vaccination were protected against a lethal challenge [47]. The specific mechanisms of protective immunization against ASFV are not yet well understood [48].

The lack of antibody development of vaccinated animals, except for one, contrast with the results of a previous study with inactivated preparation of ASFV [24], which reflected that all the animals were positive or doubtful as regards anti-ASFV p72 antibodies upon immunization, although these antibodies were not protective. Most of the animals in the study in question started to show a positive response from 7 days after boosting, while in the present study the only positive animal started to show antibodies response 14 days after boosting. The difference between the two studies could, perhaps, be explained by the fact that two different ASFV isolates were used, each of which induces a different immune response.

The animals from Group 5 succumbed one and two days before those in the control group. Moreover, these animals did not develop specific clinical signs when compared to the other animals, as described above. This suggests that the clinical evolution of the disease in the animals from Group 5, which were vaccinated without adjuvants, was slightly faster than for the controls and the remaining vaccinated groups. This slight acceleration of the disease has also been described in previous ASF immunization trials, suggesting that the enhancement of the disease is related to the development of an ineffective immune response [14,24,28].

However, the animals from Groups 1 and 3 reached the humane endpoint after the control group and with lower clinical scores (Figure 1). In addition, one of these animals (ID 2) was the only antibody-positive pig (against ASFV p72), and opposed the immune-response dependent disease enhancement, as previously suggested [14,24,28]. This shows that the adjuvants selected improved the efficacy of the inactivated preparation. Nevertheless, it should be noted that these inferences are being made with slight statistically and biologically significant differences, and with a low number of animals per group.

The negative results of this study along with the previous ones with inactivated vaccine against ASF suggests that an inactivated vaccine against ASF does not, to date, appear to be a viable strategy. The lack of efficacy of inactivated vaccines, along with the insufficiency of subunit vaccines, can be explained by the fact that cellular immunity plays a crucial role in protection against ASFV [26,49]. For a cellular response to be generated, there must be extensive viral replication in the host [50], which explains the effectiveness of live attenuated vaccines.

## 5. Conclusions

The current alarming situation of ASF worldwide signifies that a reconsideration of the containment and prevention measures taken so far is urgently required. Vaccination is a tool that could greatly help in its control, principally in the most vulnerable countries, in which sanitary measures are extremely difficult to follow owing to the lack of industrialization of the pig sector, the absence of traceability, or the low financial compensation provided to livestock farmers [51,52]. It is, therefore, essential to create an effective vaccine that could be available in a short time to fight ASFV infection. The lack of effectiveness of inactivated vaccines, as demonstrated in this work and previous studies [20,24], indicates that inactivated preparations do not appear to be a viable ASF vaccine option. Furthermore, live attenuated vaccines could be a reality in the short and medium-term, as stated previously [20,53]. Considering the virulent nature of the virus and its spread in endemic regions or regions in danger of becoming endemic [4], and in wild suid populations [13], it is vital to develop emergency vaccines. Current research efforts should, therefore, focus on clinical trials with preparations whose protective effectiveness has been proven.

## Figures and Tables

**Table 1:** Summary of the experimental design.

Group (*n* = 2)	Antigen	Dose per Route (HAD_50_) *	Adjuvant	Administration	Challenge; Dose (HAD_50_)
1	Inactivated ASFV *	6 × 10^9^	MF59^®^	2 × IM * + ID * with a 4-week interval	Pol16/DP/OUT211;10^1^
2	Inactivated ASFV	6 × 10^9^	Silica oil	2 × IM + ID with a 4-week interval	Pol16/DP/OUT211;10^1^
3	Inactivated ASFV	6 × 10^9^	mGNE	2 × IM + ID with a 4-week interval	Pol16/DP/OUT211;10^1^
4	Inactivated ASFV	6 × 10^9^	Montanide^TM^ ISA201	2 × IM + ID with a 4-week interval	Pol16/DP/OUT211;10^1^
5	Inactivated ASFV	6 × 10^9^	-	2 × IM + ID with a 4-week interval	Pol16/DP/OUT211;10^1^
Control	-	-	-	-	Pol16/DP/OUT211;10^1^

* African swine fever virus (ASFV); haemadsorption in 50% of infected cultures (HAD_50_); intramuscularly (IM); intradermal (ID).

## Data Availability

Data is contained within the article.
